# Notch1 Activation Up-Regulates Pancreatic and Duodenal Homeobox-1

**DOI:** 10.3390/genes4030358

**Published:** 2013-07-19

**Authors:** Shi-He Liu, Guisheng Zhou, Juehua Yu, James Wu, John Nemunaitis, Neil Senzer, David Dawson, Min Li, William E. Fisher, F. Charles Brunicardi

**Affiliations:** 1 Department of Surgery, David Geffen School of Medicine at University of California, Los Angeles, CA 90095, USA; E-Mails: sliu@mednet.ucla.edu (S.-H.L.); gzhou@mednet.ucla.edu (G.Z.); juehuayu@mednet.ucla.edu (J.Y.); jameswu@mednet.ucla.edu (J.W.); 2 CURE: Digestive Disease Research Center, David Geffen School of Medicine at University of California, Los Angeles, CA 90095, USA; E-Mail: ddawson@mednet.ucla.edu; 3 Mary Crowley Cancer Research Center, Dallas, TX 75230, USA; E-Mails: jnemunaitis@marycrowley.org (J.N.); nsenzer@marycrowley.org (N.S.); 4 Department of Pathology, David Geffen School of Medicine at University of California, Los Angeles, CA 90095, USA; 5 Department of Neurosurgery, UT-Houston School of Medicine, Houston, TX 77030, USA; E-Mail: min.li@uth.tmc.edu; 6 Michael E. DeBakey Department of Surgery, Baylor College of Medicine, Houston, TX 77030, USA; E-Mail: wfisher@bcm.edu; 7 Elkins Pancreas Center, Baylor College of Medicine, Houston, TX 77030, USA

**Keywords:** Notch1, NICD, PDX-1, insulin, hyperinsulinemia, hypoglycemia

## Abstract

Transcription factor pancreatic and duodenal homeobox-1 (PDX-1) plays an essential role in pancreatic development, β-cell differentiation, maintenance of normal β-cell function and tumorigenesis. PDX-1 expression is tightly controlled through a variety of mechanisms under different cellular contexts. We report here that overexpression of Notch1 intracellular domain (NICD), an activated form of Notch1, enhanced PDX-1 expression in both PDX-1 stable HEK293 cells and mouse insulinoma β-TC-6 cells, while NICD shRNA inhibited the enhancing effect. NICD-enhanced PDX-1 expression was accompanied by increased insulin expression/secretion and cell proliferation in β-TC-6 cells, which was reversed by NICD shRNA. Cre activation-induced specific expression of NICD in islet β cells of transgenic β^NICD+/+^ mice induced increased expression of PDX-1, insulin and proliferating cell nuclear antigen (PCNA) and decreased expression of p27 with accompanied fasting hyperinsulinemia and hypoglycemia and altered responses to intraperitoneal glucose tolerance test. Systemically delivered NICD shRNA suppressed islet expression of PDX-1 and reversed the hypoglycemia and hyperinsulinemia. Moreover, expression levels of NICD were correlated with those of PDX-1 in human pancreatic neuroendocrine tumor. Thus, Notch1 acts as a positive regulator for PDX-1 expression, cooperates with PDX-1 in the development of insulin overexpression and islet cell neoplasia and represents a potential therapeutic target for islet neoplasia.

## 1. Introduction

Pancreatic and duodenal homeobox-1 (PDX-1) is a homeodomain-containing transcription factor and plays an essential role in a variety of cellular processes, including pancreatic development, β-cell differentiation, maintenance of normal β-cell function and tumorigenesis. Targeted ablation of *pdx-1* gene in mice [1] and a homozygous nonsense mutation in the human *pdx-1* gene [2] results in pancreatic agenesis. PDX-1 expression is essentially restricted to the islet β-cells in adults, where it binds to the promoters of several genes essential for glucose sensing and insulin synthesis, including insulin, glucose transporter 2, and glucokinase and regulates their expression. Mice with β-cell-specific ablation of *pdx-1* develop overt diabetes [3], whereas heterozygosity for the null mutation of *pdx-1* results in decreased insulin expression/secretion [3,4] and predispose islets to apoptosis [5]. Gene mutations in human *pdx-1* lead to the development of diabetes [6]. The involvement of PDX-1 in tumorigenesis is evidenced by its overexpression in a variety of human cancers including pancreatic neuroendocrine tumor (PNET) [7,8,9,10,11,12,13] and by the significant correlation of PDX-1 overexpression with the pathological parameters of cancer patients (e.g., metastasis and histological grade) [9,14]. Recent studies have demonstrated the oncogenic properties of PDX-1 as it stimulates cell proliferation, colony formation, invasion and tumor growth [15] and is required for K-Ras^G12D^ to induce the development of PanIN, metaplasia and pancreatic ductal adenocarcinoma [16]. Moreover, PDX-1 has been shown to be a potential therapeutic target for pancreatic cancer, insulinoma and islet neoplasia [11,17].

Notch proteins are a group of transmembrane receptors, including Notch1 to Notch 4 in mammals. Notch ligands include Delta-like 1, 3 and 4 and Jagged 1 and 2 in mammals. Notch signaling is initiated by cell-to-cell interaction-mediated binding of a Notch ligand to a Notch receptor. The interaction-induced proteolytic cleavages of Notch1 generates Notch1 intracellular domain (NICD) which subsequently translocates to the nucleus and regulates the expression of a wide array of target genes such as cell cycle-related regulators (e.g., p21 and Cyclin D1) [18,19], transcription factors (e.g., c-Myc and NF-κB) [20,21] and growth factor receptors (e.g., ErbB2) [22] dependent on the cellular contexts. The highly conserved Notch signaling plays an essential role in embryonic development, cellular differentiation, proliferation and survival [23,24,25] and tumorigenesis, as an oncogene or a tumor suppressor [26,27]. The Notch signaling has also been shown to be involved in the pathology of diabetes. The pharmacological blockade of Notch signaling with inhibitors of γ-secretase, critical for the processing of the Notch proteins, raises insulin sensitivity [28]. Expression of an activated mutant of Notch (ICD-E) in both liver and small intestine in mice results in mild insulin resistance [29]. Mind bomb 1 is essential for generating functional Notch ligands to activate Notch and required for pancreatic β-cell formation [30,31]. A recent study shows that Notch signaling proteins HES-1 and Hey-1 bind to insulin degrading enzyme (IDE) proximal promoter and regulated its transcription and activity, suggesting a potential link between the Notch signaling and the expression/secretion of insulin [32]. 

PDX-1 expression is subject to positive regulation by glucose [33], GLP-1 [34,35], palmitic acid [36] and EGF [37], and negative regulation by DNA damage stimulation [38], stress [39] and SSTR5 [40,41]. Cellular expression levels of PDX-1 are tightly controlled at both transcriptional [42,43,44] and post-translational levels [38,39,45,46,47]. A number of signaling pathways have been identified to be involved in regulation of PDX-1 expression, include AKT [37], PI3K [48], PKC [49], JNK [50] and p38 [51], positively or negatively, under different cellular contexts. The purpose of this study was to determine the role of Notch1 signaling in regulation of PDX-1 expression and PDX-1-mediated cellular functions.

## 2. Results and Discussion

### 2.1. Notch1 Intracellular Domain (NICD) Enhances PDX-1 Expression in PDX-1 Stable HEK 293 Cells and β-TC-6 Cells

**Figure 1:**
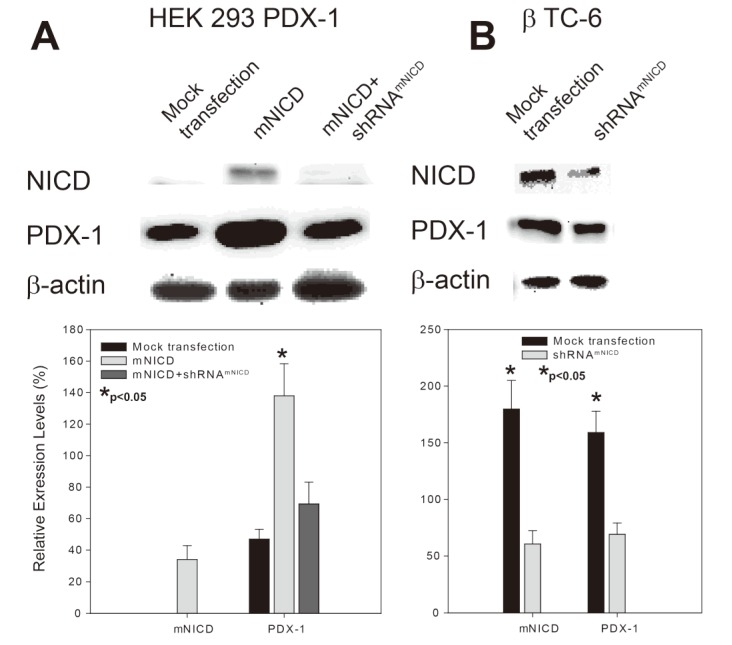
NICD up-regulates PDX-1 expression in PDX-1 stable HEK293 and β-TC-6 cells. (**A**) pcDNA^mNICD^ with shRNA^mNICD_scrambled^ or shRNA^mNICD^ were co-transfected into PDX-1 HEK293 cells using Lipofectamine 2000. (**B**) shRNA^mNICD^ was transfected into β-TC-6 cells using Lipofectamine 2000. The cells were collected 40 hours after transfection. Whole cell lysates were subjected to SDS-PAGE, followed by Western blotting with an anti-NICD, -PDX-1 and β-actin antibody. Upper panels are representative Western blotting experiments. Lower panels are the densitometric analysis of three independent Western blotting experiments (∗ indicates *p* < 0.05 showing significant difference).

To determine the role of Notch1 signaling in regulation of PDX-1 expression, we transfected Notch1 intracellular domain (NICD), activated form of Notch1, alone or NICD plus NICD shRNA (shRNA^NICD^) into PDX-1 stable HEK293 cells [15]. Western blotting analysis showed that overexpression of NICD resulted in a significant increase of PDX-1 expression compared to that of mock transfection (Figure 1A, column 2 *vs.* column 1), while co-transfection of shRNA^NICD^ with NICD only resulted in a mild increase of PDX-1 expression (Figure 1A, column 3 *vs.* column 1). These data indicate that NICD, activated Notch1, acts as a positive regulator for PDX-1 expression and that NICD shRNA inhibits the enhancing effect of NICD on PDX-1 expression. To confirm the positive regulation of PDX-1 expression by Notch1 activation, we examined the effect of NICD shRNA (shRNA^NICD^) on endogenous PDX-1 expression in mouse insulinoma β-TC-6 cells which have high expression level of PDX-1 [41]. shRNA^NICD^ was transfected into β-TC-6 cells. Western blotting analysis showed that NICD was abundant in β-TC-6 cells and transfection of shRNA^NICD^ resulted in a significant knockdown of endogenous NICD (Figure 1B, top panel). In parallel, PDX-1 expression level was also significantly decreased by transfection of shRNA^NICD^ in comparison to the mock transfection (Figure 1B, middle panel), further supporting the concept that activated Notch1 (NICD) is a positive regulator for PDX-1 expression.

The underlying mechanism by which Notch1 activation up-regulates PDX-1 expression is not known. It has been well established that, upon ligand binding, NICD is released from the Notch protein and moves to the nucleus, where it regulates gene expression by interacting with and activating transcription factors such as CBF1/RBP-Jκ/Suppressor of Hairless/LAG-1 (CSL) transcription factor and p300 [52,53]. Given that PDX-1 is a potent transcription factor for PDX-1 itself, it is, thus, possible that NICD may up-regulate the transcriptional activity of PDX-1, which, in turn, enhances the expression of PDX-1. However, we cannot exclude the possibility that NICD might enhance PDX-1 expression by up-regulating other transcription factors, which target PDX-1. Further studies warrant a better understanding of the molecular basis for the positive regulation of PDX-1 expression by Notch1 activation.

### 2.2. Notch1 Activation-Enhanced PDX-1 Expression Is Accompanied by Increased Insulin Expression/Secretion and Cell Proliferation in β-TC-6 Cells

**Figure 2:**
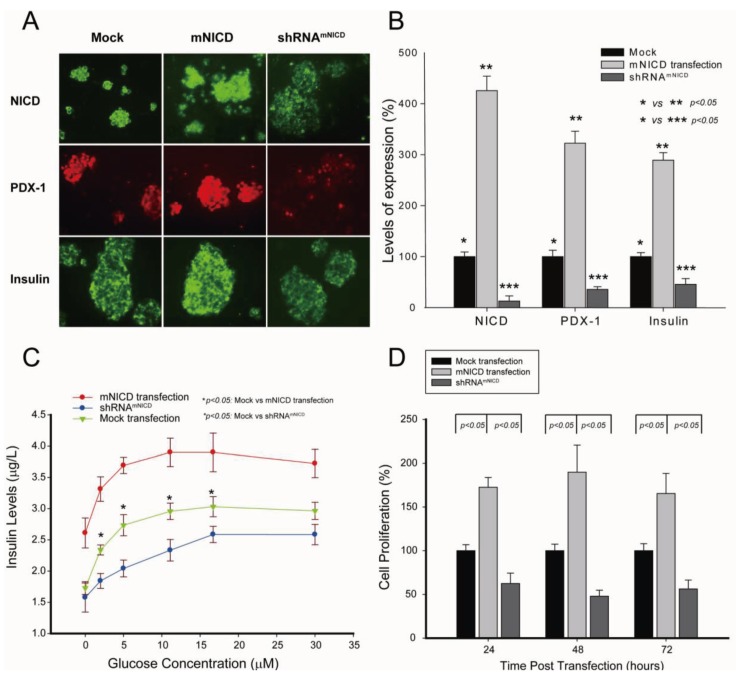
NICD-enhanced PDX-1 expression is accompanied by increased insulin expression/secretion and cell proliferation in β-TC-6 cells. mNICD or shRNA^mNICD^ was transfected into β-TC-6 cells using Lipofectamine 2000. (**A**) Immunohistochemistry analyses were performed using antibodies against NICD, PDX-1 (1:200) or insulin (1:75). Fluorescence was developed using cy3- or FITC-conjugated secondary antibody. Photomicrographs were taken under 100× magnification. (**B**) Immunostaining for the expression of NICD, PDX-1 and insulin was semi-quantified using ImageJ (∗ indicates *p* < 0.05 showing significant difference). (**C**) Forty-eight (48) hours after transfection, β-TC-6 cells were washed twice with Krebs-Ringer bicarbonate (KRB) buffer and incubated in KRB-BSA for 1 hour. The cells were then added a variety of concentrations of glucose as indicated for 4 hours. After incubation, the media were collected and centrifuged at 600 g for 5 minutes. The insulin concentrations in the media were measured by ELISA assay (∗ indicates *p* < 0.05: Mock *vs.* mNICD transfection; *p* < 0.05: Mock *vs.* shRNA^mNICD^ transfection, showing significant difference). (**D**) Twenty four (24) hours after transfection, β-TC-6 cells were replated into 96-well cell culture plates at 5 × 10^3^ cells/well. Cell proliferation was determined by MTS assay (Promega, Madison, WI, UAS) at 24, 48, and 72 hour after transfection. The absorbance was read using a Multiskan EX plate reader (Thermo Electronic Corp, Franklin, MA, USA) at 492 nm.

PDX-1 plays an essential role in insulin expression and secretion [3,4] and cell proliferation [54]. To determine the functional relevance of the positive regulation of PDX-1 by NICD, we sought to examine the effect of NICD on PDX-1-mediated insulin expression/secretion and cell proliferation in β-TC-6 cells. β-TC-6 cells were transfected with NICD or shRNA^NICD^. Immunohistochemistry analysis showed that overexpression of NICD further increased NICD expression in β-TC-6 cells, while overexpression of shRNA^NICD^ inhibited NICD expression (Figure 2A, top panel). As expected, PDX-1 expression level was increased by transfected NICD and decreased by transfected shRNA^NICD^ (Figure 2A, middle panel). Moreover, we found that NICD-enhanced PDX-1 expression was accompanied by increased insulin expression, and NICD knockdown-induced inhibition of PDX-1 expression was accompanied by decreased insulin expression (Figure 2A, bottom panel). Insulin ELISA analysis showed that NICD overexpression significantly increased glucose stimulated insulin secretion (GSIS) in comparison to mock transfection, whereas shRNA^NICD^ led to significant inhibition of insulin secretion in β-TC-6 cells (Figure 2B). By performing MTS assays, we found that, at 24, 48 and 72 hour after transfection, overexpression of NICD resulted in significant increases of β-TC-6 cell proliferation by 174 ± 18.2%, 185 ± 30.8% and 166 ± 21.3%, respectively, as compared to the mock transfection (Figure 2C). Conversely, transfection of shRNA^NICD^ led to significant decreases of cell proliferation by 63 ± 15.3%, 49 ± 8.8% and 56 ± 12.6% at each time point (Figure 2C). All these data indicate that Notch1 activation exerts a positive effect on insulin expression/secretion and cell proliferation likely through a mechanism of up-regulating PDX-1 expression in β-TC-6 cells.

### 2.3. Notch1 Activation Induces Hypoglycemia and Hyperinsulinemia in a Transgenic β^NICD+/+^ Mouse Model

**Figure 3:**
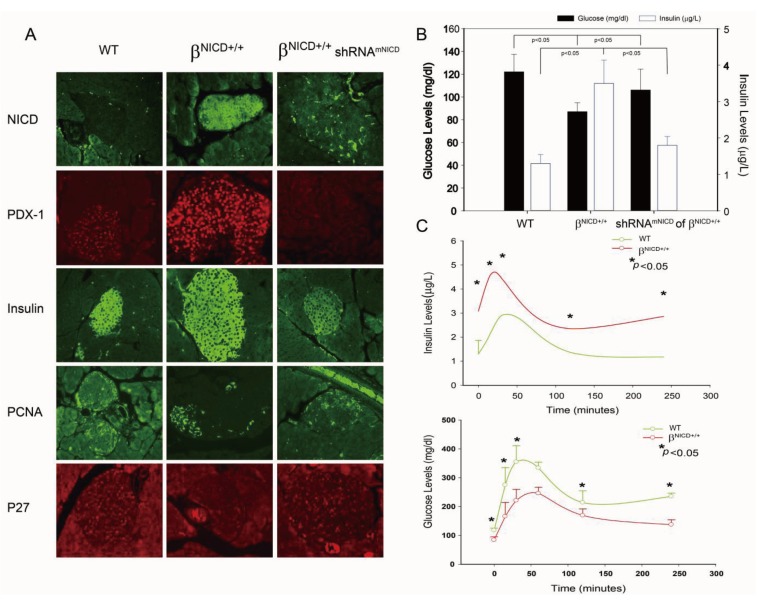
Overexpression of NICD-induced increase of PDX-1 expression is accompanied by the development of hypoglycemia and hyperinsulinemia in a β^NICD+/+^ transgenic mouse model. (**A**) Whole pancreatic tissues isolated from control mice (WT), β^NICD+/+^ mice and β^NICD+/+^ treated with shRNA^mNICD^ were fixed in 4% (v/v) paraformaldehyde and embedded in paraffin. Immunohistochemistry analyses were performed using antibodies against NICD, PDX-1 (1:200), insulin (1:75), PCNA and p27. Fluorescence was developed using cy3- or FITC-conjugated secondary antibody. Photomicrographs were taken under 100× magnification. (**B**) Fifty (50) μL of whole blood samples were collected from control mice (WT), β^NICD+/+^ mice and β^NICD+/+^ mice treated with shRNA^mNICD^ and spun to separate the serum. Glucose levels were measured using a Beckman-Coulter Glucose Analyzer 2 (Coulter-Beckman, Fullerton, CA, USA), and presented as mean ± S.E.M. in mg/dl. Insulin levels were determined using a mouse insulin ELISA kit from Mercodia (Linco Research, St. Charles, MO, USA) and presented as mean ± S.E.M. in μg/L. (**C**) Control mice (WT) and β^NICD+/+^ mice at age of 6 months were fasted 6 hours before collection of blood samples as T_0_. Grouped mice were then given 1.2 g glucose/kg body weight via *ip* injection, followed by collection of blood samples at 15, 30, 60, 120 and 240 minute after injection of glucose. Glucose and insulin levels were measured as described above.

To further confirm the up-regulation of PDX-1 expression by Notch1 activation, we generated a transgenic β^NICD+/+^ mouse model by crossing LSL-ROSA^NICD^ mice [55] and RIP1-Cre mice, which resulted in β cell-specific expression of NICD within the islets. To determine the effect of NICD on PDX-1 and PDX-1-mediated cellular functions, the β^NICD+/+^ mice were treated at six months of age with or without liposomal shRNA^mNICD^ at a dose of 35 μg per mouse via tail vein injection. The mice tolerated the treatment well with no toxic effects observed. Forty eight (48) hours after treatment of shRNA^mNICD^, the mice were sacrificed and the pancreata were isolated, fixed and prepared for immunohistochemistry analysis using anti-NICD, PDX-1, insulin, PNCA and p27 antibodies, respectively. As shown in Figure 3A, Cre activation induced a significant increase of NICD expression in islet cells of β^NICD+/+^ mice as compared to that of control mice, and systemically-delivered shRNA^mNICD^ efficiently knocked down NICD (top panel). Cre activation-induced overexpression of NICD was accompanied by increased expression of PDX-1, insulin and proliferating cell nuclear antigen (PCNA) and a decreased expression of p27, an inhibitor of cyclin-dependent protein kinase, in islet cells of β^NICD+/+^ mice as compared to those of control mice. This is consistent with the finding that Notch directs the transcription of the E3 ubiquitin ligase S phase kinase-associated protein 2 (SKP2), which results in a decrease of p27 protein levels and an increase of cell proliferation [56]. In contrast, knockdown of NICD by systemically-delivered shRNA^mNICD^ resulted in reduction of the expression of PDX-1, insulin and PCNA and increased expression of p27. These *in vivo* data further support the concept that Notch1 activation up-regulates insulin expression/secretion and cell proliferation through a mechanism involving enhancing PDX-1 expression. We noticed a previous study that PDX-1 expression is not affected by NICD in both *Rosa^N^°^tch/+^*;*Pdx1-Cre* and *Rosa^N^°^tch^;Pdx1-CreER* mice [55]. The discrepancy may be due to the different Cre systems used (*Pdx1-Cre* and *Pdx1-CreER vs.* *RIP1-Cre*). In addition, the mice we analyzed were six months old, while the mice Murtaugh *et al.* analyzed were newborn or embryo [55].

Given the essential role of PDX-1 in regulation of insulin expression/secretion, we hypothesized that NICD-induced increase of PDX-1 expression may lead to the development of hypoglycemia and hyperinsulinemia in β^NICD+/+^ mice. To test this hypothesis, glucose and insulin levels were measured in β^NICD+/+^ mice at six months of age. We found that glucose levels were significantly decreased and insulin levels were significantly increased in β^NICD+/+^ mice compared to those in control mice, while treatment of the β^NICD+/+^ mice with shRNA^mNICD^ rescued/restored glucose and insulin levels close to wild type levels (Figure 3B).

Next, we performed IPGTT in control (WT) and β^NICD+/+^ mice at six months of age to compare glucose tolerance. Basal insulin levels in β^NICD+/+^ mice were significantly higher than those in control mice (Figure 3C, top panel), whereas fasting glucose levels in β^NICD+/+^ mice were significantly lower than those in control mice (Figure 3C, bottom panel). Following intraperitoneal glucose injection, systemic insulin levels in β^NICD+/+^ mice were significantly higher than those in control mice at 15, 30, 120 and 240 minute post-injection (Figure 3C, top panel). Glucose levels in β^NICD+/+^ mice were significant lower than those in control mice at 15, 30, 60, 240 minute post injection of glucose (Figure 3C, bottom panel). These data indicate that overexpression of NICD resulted in fasting hypoglycemia and hyperinsulinemia and alterations in insulin and glucose responses to IPGTT.

Taken together, our studies show that NICD-enhanced PDX-1 expression was accompanied by increased insulin expression/secretion and cell proliferation in β cells of transgenic β^NICD+/+^ mice, resulting in hyperinsulinemia and hypoglycemia, as well as an altered response to IPGTT in these mice. A single systemic treatment with liposomal NICD shRNA resulted in *in situ* knockdown of NICD and PDX-1, leading to reversal of hyperinsulinemia and hypoglycemia. Moreover, there were no overt toxic side effects following the single treatment of liposomal NICD shRNA. This leads further support that Notch1 regulates PDX-1 expression and suggests that Notch1 could be a potential therapeutic target for hypoglycemic disorder using an RNAi platform. This is consistent with our recent studies showing that three-cycle treatment of PDX-1 shRNA reverses hyperinsulinemia and hypoglycemia in an immune-competent mouse model of islet neoplasia and results in significant reduction of tumor volume and improved survival in a human pancreatic cancer xenograft mouse model [11]. Our results also show that the transgenic β^NICD+/+^ mice with conditional induction of NICD in islet cells may serve as a potential novel mouse model for the development of islet cell neoplasia. 

### 2.4. Activation of Notch1 Is Associated with PDX-1 Overexpression in Human Pancreatic Neuroendocrine Tumors (PNETs)

**Figure 4:**
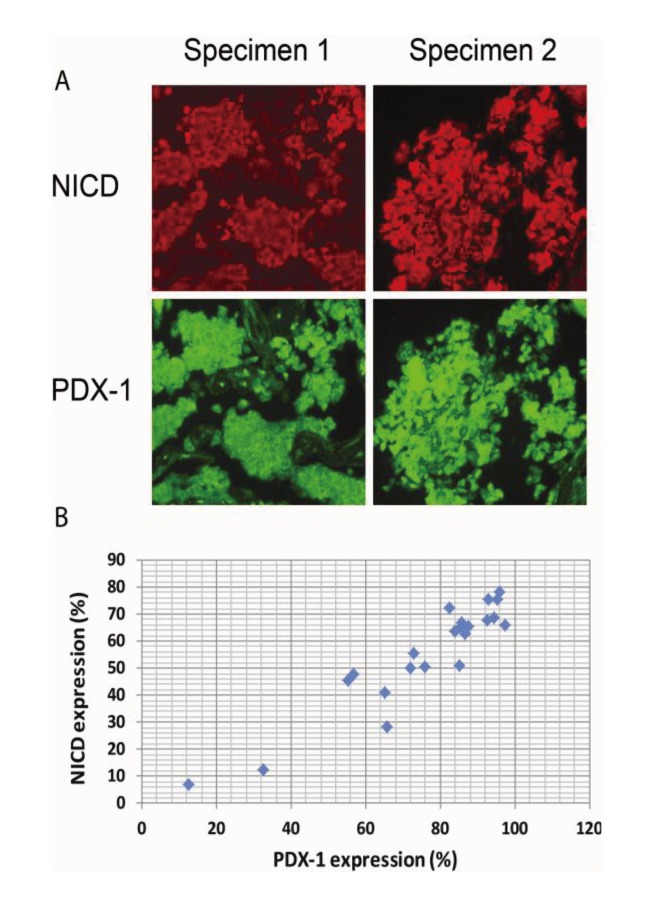
Activation of Notch1 is associated with PDX-1 overexpression in human PNETs.Human PNET specimens were fixed in 4% (v/v) paraformaldehyde and embedded in paraffin. After deparaffinization in xylene and rehydration through graded alcohol, tissue sections were incubated with an anti-NICD (1:100) or an anti-PDX-1 antibody (1:200) overnight at 4 °C. Fluorescence was developed using FITC- or Cy3-conjugated secondary antibody. Simultaneous fluorescence microscopy observation and photography were carried out using an Olympus IX70 microscope (200×). (**A**) Shown are representative micrographs showing the expression levels of NICD and PDX-1 in PNET specimens. (**B**) Pearson correlations analysis showed significant correlation between PDX-1 and NICD expression in these specimens, R = 0.933; *p* < 0.01.

It has been reported that Notch1 is over-expressed in neuroendocrine tumors. We have recently shown that PDX-1 is markedly overexpressed in PNETs [41]. We, therefore, wanted to examine whether Notch1 activation is correlated with PDX1 overexpression in PNETs by performing IHC analysis of 35 human PNET specimens. We found that Notch1 was activated in 31 of the 35 specimens (89%) as evidenced by NICD expression and that PDX-1 was expressed in all PNET specimens studied. Semi-quantitative analysis of the immunofluorescence images for NICD and PDX-1 using software ImageJ revealed that the median expression levels of PDX-1 were 85.2% and that of NICD was 63.8%. Moreover, we found that higher expression levels of NICD were accompanied by higher expression levels of PDX-1 (Figure 4A, right panel *vs.* left panel). Pearson correlations analysis showed significant correlation between PDX-1 and NICD expression (Figure 4B, R = 0.933; *p* < 0.01). These data further support the concept that Notch1 activation up-regulates PDX-1 expression and that Notch1 activation may contribute to the overexpression of PDX-1 in PNETs. Notch1 has been reported to be expressed in PNET with accompanied expression of HES1, a downstream target of Notch1 [57]. Our studies showed that the majority of human PNET specimens expressed NICD in correlation with PDX-1 overexpression. Moreover, we found that NICD-enhanced PDX-1 expression was accompanied by increased cell proliferation in mouse insulinoma β-TC-6 cells and resulted in increased PCNA in the islets of transgenic β^NICD+/+^ mice. Given the oncogenic function of PDX-1 [15], our study suggests that Notch1 may play an oncogenic role in PNETs via up-regulating PDX-1. Cooperation with other oncogenes is one mechanism by which Notch1 exerts its oncogenic function. Our studies, thus, provide that up-regulation of PDX-1 is one novel mechanism by which Notch1 exerts its oncogenic functions.

## 3. Experimental Section

### 3.1. Cell Lines, Vectors, Antibodies and PNET Specimens

Human embryonic kidney 293 (HEK 293) and mouse insulinoma β TC-6 cells were purchased from the American Type Culture Collection (ATCC, Bethesda, MD, USA) and maintained in Dulbecco’s modified Eagle medium (Gibco-BRL, Bethesda, MD, USA) supplemented with 100,000 U/L of penicillin, 100,000 µg/L of streptomycin, and 10% fetal bovine serum (FBS). pBS-Notch1-IC (mouse) was purchased from Addgene (plasmid 15079) and subcloned into pcDNA3.1 expressing vector to produce pcDNA^mNICD^. Human PDX-1 cDNA was polymerase chain reaction (PCR)-amplified from human islet cells and cloned into the FLAG epitope containing pCMV5 expression vector which was provided by Dr. Narasimhaswamy Belaguli (Baylor College of Medicine). Both mouse Notch1-IC and human PDX-1 were subcloned into retroviral vector pQICXIP (Clontech, Mountain View, CA, USA) for the purpose of retrovirus production. Five mouse Notch shRNAs targeting NICD (shRNA^mNICD^) were designed, synthesized and cloned into pSuper vectors. One of them targeting AGGCAACAGTGAAGAAGAA (starting 5661 of open reading frame of Notch1) was selected for the current study based on its most efficient knockdown effect. A scrambled shRNA was used as control. Goat anti-PDX-1 and rabbit anti-p27 and anti-PCNA antibodies were purchased from Santa Cruz Biotechnology Inc (Santa Cruz, CA, USA). Rabbit anti-activated Notch1 antibody that only recognizes the cleaved intracellular (activated) form of Notch1 (NICD) was purchased from Abcam (Cambridge, MA, USA). Monoclonal anti-insulin antibody was purchased from Sigma-Aldrich (St. Louis, MO, USA). Goat anti-rabbit antiserum and sheep anti-mouse antiserum conjugated with horseradish peroxidase were purchased from Amersham Life Science Inc. (Arlington Heights, IL, USA). Rabbit anti-goat IgG was obtained from Zymed Laboratories Inc. (South San Francisco, CA, USA). PNET specimens were as previously described [58].

### 3.2. Transient and Stable Transfection

Transient transfection in HEK293 and β-TC-6 cells was performed with 24 µg of plasmid DNAs using Lipofectamine 2000 (Invitrogen, Carlsbad, CA, USA). Stable cell lines were established using retroviral expression system with pQCXIP retroviral vectors (Clontech, Mountain View, CA, USA). pQCXIP expressing PDX-1 was constructed and confirmed, followed by transfection into the AmphoPack 293 cell line (Clontech, Mountain View, CA, USA) with Lipofactamine 2000 to produce retrovirus. The cells were infected with the supernatant containing the reconstructed retrovirus and selected with 10 mg/mL puromycin for two weeks. pcDNA^mNICD^ with shRNA^mNICD^ or shRNA^mNICD_scrambled^ were co-transfected into β-TC-6 cells.

### 3.3. Cell Proliferation Assays

Twenty-four hours after transfection, cells were replated into 96-well cell culture plates at 5 × 10^3^ cells/well. Cell proliferation was determined by MTS assay (Promega, Madison, WI, USA) at 24, 48, and 72 hour after transfection. The absorbance was read using a Multiskan EX plate reader (Thermo Electronic Corp, Franklin, MA, USA) at 492 nm. Levels of proliferation were determined according to the following formula: percentage of proliferation = 100 × B/A, where A is the absorbency at 492 nm of control cells, and B is the absorbency at 492 nm of treated cells.

### 3.4. Western Blotting

Transfections were performed at 10-cm plates for 48 hours as described as previously. Cells were collected using a cell scrapper in an ice-cold phosphate-buffered saline (PBS) solution containing 5 mM EDTA. Cells were lysed in a buffer containing 20 mM HEPES (pH 7.4), 2 mM EGTA, 50 mM β-glycerophosphatase, 1% Triton X-100, 10% glycerol, 1 mM DTT, 2 mg/mL of leupeptine, 5 mg/mL of aprotinin and 1 mM phenylmethylsulphonyl fluoride after two washes with PBS. Supernatants were collected after centrifugation at 100,000 g for 15 minutes at 4 °C. Twenty micrograms of cell lysates were analyzed using a sodium dodecyl sulfate-polyacrylamide gel electrophoresis gel (SDS-PAGE). After electrophoresis, gels were removed and blotted onto polyvinylidene fluoride (PVDF) membranes. Membranes were then incubated with 5% non-fat milk buffer and probed with antibodies against NICD and PDX-1. Immunocomplexes were visualized by enhanced chemiluminescence detection using horseradish peroxidase-conjugated secondary antibodies. Images were captured using the UVP imaging system, and the band was analyzed using ImageJ software.

### 3.5. Generation of β^NICD+/+^ Mice

LSL-Rosa^NICD^ transgenic mice were kindly provided by Dr. Brendan Lee (Baylor College of Medicine, Houston, TX, USA) with permission of use from Dr. Douglas Melton (Harvard University, Boston, MA, USA) [55]. The mice were maintained on a hybrid 129× C57BL/6 background. β^NICD+/+^ mice were generated by crossing LSL-Rosa^NICD^ and RIP1-Cre mice with successive generation screening. The β^NICD+/+^ mice at age of 6 months were treated with intravenous liposomal shRNA^mNICD^ at dose of 35 μg per mouse via tail vein injection. Forty-eight (48) hours after injection, blood was collected and mice were sacrificed for tissues collection. Animals were used in accordance with the National Institutes of Health Guide for the Care and Use of Laboratory Animals. All mice were housed in a specific pathogen-free facility and under light-, temperature-, and humidity-controlled conditions. These studies were performed under IRB approval.

### 3.6. Insulin and Glucose Measurements

50 μL whole blood samples were collected and spun to separate the serum. Serum samples were stored at −20 °C until completion of experiments. Glucose levels were measured using a Beckman-Coulter Glucose Analyzer 2 (Coulter-Beckman, Fullerton, CA, USA), and presented as mean ± S.E.M. in mg/dL. Insulin levels were determined using a mouse insulin ELISA kit from Mercodia (Linco Research, St. Charles, MO, USA) and presented as mean ± S.E.M. in μg/L.

### 3.7. Intraperitoneal Glucose Tolerance Test (IPGTT)

Control (WT) and β^NICD+/+^ mice at age of six months were fasted 6 hours before collection of blood samples as T_0_. Grouped mice were then given 1.2 g glucose/kg body weight via ip injection, followed by collection of blood samples at 15, 30, 60, 120 and 240 minute after injection of glucose. Glucose and insulin levels were measured as described above.

### 3.8. Immunohistochemical Staining

Fluorescein isothiocyanate-conjugated anti-rabbit IgG antibodies were purchased from Sigma (St. Louis, MO, USA). PNET tumors were fixed in 4% paraformaldehyde at 4 °C overnight. After treatment with 70% alcohol, tissue blocks were embedded in paraffin, and tissue sections were prepared. For immunostaining, sections were deparaffinized in xylene and hydrated gradually through graded alcohol. Slides were then placed in a humidified chamber, overlaid with diluted antibodies (1:100) against activated Notch1, PDX-1, insulin, PCNA or p27, and incubated overnight at 4 °C. After washing with PBS, slides were incubated with FITC-conjugated anti-rabbit or Cy3-conjugated anti-goat secondary antibody for 1 hour, then washed with PBS and mounted with cover slides. Images were recorded using a digital camera (Diagnostic Instruments Inc., Sterling Heights, MI, USA) on a fluorescent microscope (Olympus IX70; Olympus Optical Co Ltd., Japan). Immunostaining for PDX-1 expression was semi-quantified using ImageJ.

### 3.9. Statistical Analysis

Student’s t-test was used to analyze difference in means of the continuous data. All numeric data are expressed as mean ± SEM, with *p* < 0.05 indicating significance. Pearson correlation analysis was used to analyze the correlation between PDX-1 and NICD expression

## 4. Conclusions

In this study, we provided biochemical and genetic evidence showing that Notch1 activation plays a positive role in regulation of PDX-1 expression as evidenced by the increased level of PDX-1 in PDX-1 stable HEK293 cells, β-TC-6 cells and in β cells of transgenic β^NICD+/+^ mice in the presence of NICD, a activated form of Notch1. We also found that NICD-enhanced PDX-1 expression was accompanied by increased insulin expression/secretion and cell proliferation in β-TC-6 cells and in islets of β^NICD+/+^ mice. β^NICD+/+^ mice were hyperinsulinemic and hypoglycemic, suggesting that Notch1 activation results in islet neoplasia. Systemically-delivered NICD shRNA reversed the enhancing effect of NICD on PDX-1, as well as on hyperinsulinemia and hypoglycemia, suggesting that Notch1 could be a potential therapeutic target. Thus, Notch1 may exert an oncogenic function in the development of islet cell neoplasia through cooperation with PDX-1.
